# Geometric Structure of 3D Spinal Curves: Plane Regions and Connecting Zones

**DOI:** 10.5402/2012/840426

**Published:** 2012-02-20

**Authors:** E. Berthonnaud, R. Hilmi, J. Dimnet

**Affiliations:** ^1^Hôpital Nord Ouest site de Villefranche, BP436, 69655 Villefranche Saone, France; ^2^Laboratoire de Physiologie de l'Exercice, Université de Lyon, 42023 Saint Etienne, France; ^3^Group of Applied Research in Orthopaedic, 69005 Lyon, France

## Abstract

This paper presents a new study of the geometric structure of 3D spinal curves. The spine is considered as an heterogeneous beam, compound of vertebrae and intervertebral discs. The spine is modeled as a deformable wire along which vertebrae are beads rotating about the wire. 3D spinal curves are compound of plane regions connected together by zones of transition. The 3D spinal curve is uniquely flexed along the plane regions. The angular offsets between adjacent regions are concentrated at level of the middle zones of transition, so illustrating the heterogeneity of the spinal geometric structure. The plane regions along the 3D spinal curve must satisfy two criteria: (i) a criterion of minimum distance between the curve and the regional plane and (ii) a criterion controlling that the curve is continuously plane at the level of the region. The geometric structure of each 3D spinal curve is characterized by the sizes and orientations of regional planes, by the parameters representing flexed regions and by the sizes and functions of zones of transition. Spinal curves of asymptomatic subjects show three plane regions corresponding to spinal curvatures: lumbar, thoracic and cervical curvatures. In some scoliotic spines, four plane regions may be detected.

## 1. Introduction

Sagittal balance of asymptomatic subjects and patients has been studied from sagittal radiographic images [1–3]. Spinal curves show generally three regions with opposite curvatures: lumbar, thoracic, and cervical curvatures. Adjacent regional curves are bounded either by specific vertebrae in case of clinical applications or by points of inflexion when the sagittal curve is represented by a mathematical function [4, 5]. Angles of regional curvatures (Cobb angles) may be directly measured upon radiographic images or calculated from the sagittal curve equation.

The mathematical equation of the sagittal spinal projection has been obtained from using a new technique [6]. The projected spinal structure is delimited by two border curves. Each of them is represented by a B-spline curve passing a restricted number of points recorded along the border. The sagittal spinal curve representing the beam projection is defined as the median curve drawn between the two border lines. It is modeled by a B-spline curve. In sagittal projections, each spinal region showing homogeneous curvature is described by parameters defining its shape and orientation [7]. The technique defined for sagittal radiographic projections has been extended to frontal images of scoliotic spines [8], frontal and sagittal radiographic images being shot independently.

The photogrammetric technique reconstructs points in space from their two images in projection plane. The photogrammetry applied to radiographic images has been described by Suh [9]. The photogrammetric reconstruction of spinal curves from simultaneous biplanar radiography has been first presented by Brown et al. [10].

The biplanar radiography involves the setting of specific devices in case of simultaneous exposures [11–13]. Successive exposures have been used for clinical applications. They need a standard radiographic system with one X-ray source and one plate. A rotating plateau is interposed between X-ray source and plate. The patient stands motionless upon the plateau. Patient and plateau are rotated for successive frontal and sagittal radiographic images [14]. On frontal and sagittal projections median spinal curves are drawn. Points of frontal and sagittal curves are related together for the photogrammetric reconstruction of the 3D spinal curve. The relation is based on the use of epipolar planes [15]. 3D spinal curves may be projected in fixed plane. For clinical applications, three projections are available: upper view and frontal and sagittal projections for validating the technical procedure through the comparison between spinal curve projections and radiographic images.

The goal of this paper is to study the geometric structure of spinal curves defined from a series of points reconstructed from photogrammetric techniques. Stagnara et al. [16] demonstrated that scoliotic spines show highest curvatures when shot under specific radiographic incidences. A plane of maximum curvatures of scoliotic spines has been determined from points of spinal curve [14]. This technique was based on the determination of one plane located so that linear distances between 3D spinal curve and plane would be minimum. The maximum distance value governs the relevance of the geometric model representing the spinal curve as a unique plane. If the maximum distance value is too high (up to 10 mm) the unique plane model is maladapted.

The principle of local planes fitted to regions of the 3D rough spinal curve has been retained. Two criteria must be satisfied locating these planes: (i) the maximum distance between the 3D curve facing the regional plane and the unknown plane must be low (2 mm) and (ii) the regional curve must be continuously plane without local anomalies. A great number of spinal curves have an heterogeneous structure. They show plane regions, where the spine curve is purely flexed, and zones of transition between adjacent plane regions. The plane regions often correspond to lumbar, thoracic, and cervical curvatures. In asymptomatic subjects the regional planes are oriented closely. They may be strongly spreaded in patients with spinal deformities. Examples are shown for illustrating the method. Results are presented and discussed.

## 2. Materials

Frontal and sagittal images of subjects and patients have been obtained from two different radiographic systems.

- New set-up shooting simultaneously from frontal and sagittal incidences. 125 members of paramedical staffs of a local hospital accepted a biplanar radiographic examination in the frame of a clinical research studying relations between spinal features and chronic low back pain.
- A standard radiographic system completed by a rotating plateau has been set in an hospital of Lyon specialized in back pathologies. All patients having back deformities are submitted to a biplanar radiographic examination with successive X-rays with the objective of following the 3D spinal feature evolutions versus time. The access to the geometric structure of spinal curves at different steps of their evolution brings new information for clinicians.

## 3. Methods

### 3.1. The Detection of Plane Regions along the Spinal Curve

The detection is based upon two criteria: the criterion of proximity and the criterion of continuous regional planeity.

- The criterion of proximity to a plane is satisfied when all points of a local section of the rough spinal curve are close to the unknown regional plane. The maximum distance between rough curve and the facing plane is very low (2 mm). Accuracy reconstructing points using biplanar radiography coupled with photogrammetry is 1 mm.
- The criterion of continuous regional planeity controls that the plane region is continuously plane. Series of three points, located close together along the spinal curve are successively considered. The normal vector to the local plane passing through the three points is calculated. The bundle of normal vectors to the regional curve is defined. This bundle must be strongly concentrated. Aberrant points along the spinal curve introduce aberrant normal vector directions. These points are eliminated. The direction of the median normal vector is calculated from the bundle of normal vectors. Limit points bounding the plane region are then determined.

Limit points bounding each plane region are located along a diagram representing the unrolled spine from the L_5_ lower plate center to the C_3_ upper plate center. Each vertebra and each intervertebral disc are located along the unrolled spine using normalized values defined statistically [8]. A correlation is defined between the position of limit points along the unrolled spine and the level of vertebrae or discs.

### 3.2. Parametring the Orientation of the Normal Vectors to Regional Planes with Respect to the Fixed Set of Axis

The axes of the fixed frame *F*_0_(*OX*_0_ *Y*_0_ *Z*_0_) are: *OZ*_0_ vertical axis, *OX*_0_ posteroanterior axis and *OY*_0_ transverse axis. The normal vector *n*_*i*_ to the regional plane *π*_*i*_ is defined by its components *n*_*ix*_, *n*_*iy*_, *n*_*iz*_ in frame *F*_0_. The direction *n*_*i*_ might be referred to *F*_0_ using the standard technique of Euler angles. This technique involves elementary rotations about moving axes. A sequence of two rotations about fixed axes has been preferred. The normal vector *n*_*I*_ is moved from *Y*_0_ by two successive rotations: an axial rotation *ψ* about *Z*_0_ and a lateral flexion *θ* about

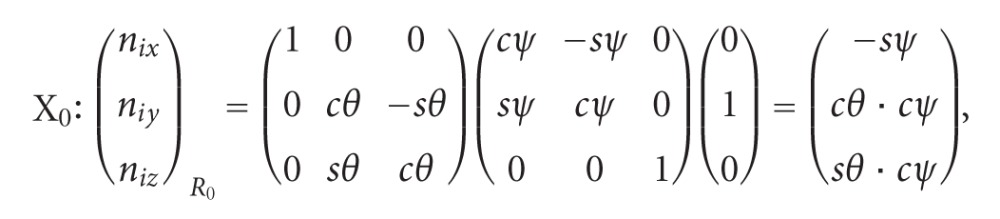
(1)
*θ* and  *ψ* are defined by the following relations:

(2) $$\begin{array}{c} \theta =\text{ta}{\text{n}}^{-1}\left(\frac{n_{iz}}{n_{iy}}\right),\\ \psi =-\text{ta}{\text{n}}^{-1}\;\left(\frac{n_{ix}\cdot c\theta }{n_{iy}}\right)\;\text{or}\;-\text{ta}{\text{n}}^{-1}\left(\frac{n_{ix}\cdot s\theta }{n_{iz}}\right). \end{array}$$

This technique introduces rotations about fixed axes. The direct comparison between orientations of the different plane regions does not depend on the position of the moving axes involved by the Euler calculation.

### 3.3. Parameters Characterizing the Geometric Pattern of the 3D Spinal Curve

Spinal curves show generally three (or more) plane regions. The planes *π*_i_ are defined, with respect to the fixed frame *OX*_0_ *Y*_0_ *Z*_0_, by the position of their origin *S*_*i*_ (starting point of the plane region) and by the two angles *ψ*_*i*_ and *θ*_*i*_ locating the vector *n*_*i*_, normal to *π*_*i*_. All points *M*_*i*_ of the spinal curve Γ_*i*_, facing *π*_*i*_, are projected in *m*_*i*_ on *π*_*i*_. The plane curve *γ*_*i*_ passing all points *m*_*i*_ is then obtained.

The geometric structure of the spinal curve is heterogeneous. It has several plane regions *π*_1_, *π*_2_, *π*_3_ of normal vectors *n*_1_, *n*_2_, *n*_3_, where the 3D curve  Γ is very close to the projected curves *γ*_1_, *γ*_2_, *γ*_3_. Each plane curve *γ*_*i*_ is purely flexed. Zones of transitions between sacrum and,  *π*_1_ between *π*_1_ and *π*_2_, and between *π*_2_ and *π*_3_ are characterized by angular offsets between normal vectors *Y*_0_ and *n*_1_, *n*_1_ and *n*_2_, and *n*_2_ and *n*_3_. These angular offsets include axial rotations and abductions needed for moving the sagittal plane *OxO*_*Z*_ to *π*_1_, *π*_1_ to *π*_2_, *π*_2_ to *π*_3_.

The plane curves *γ*_*i*_ are characterized by values of parameters describing their shape [14].

- Positions of limit points starting points *S*_*i*_ and arrival *A*_*i*_ bounding the plane region *π*_*i*_.
- Length *ρ*_*i*_ and orientation *α*_*i*_ of the linear segment *S*_*i*_ *A*_*i*_.
- Position of *K*_*i*_ point of maximum regional offset referred to the segment *S*_*i*_ *A*_*i*_. The tangent in *K*_*i*_  to *γ*_*i*_  is parallel to *S*_*i*_ *A*_*i*_.
- Angles *t*_*si*_ and *t*_*Ai*_ included between segment *S*_*i*_ *A*_*i*_ and tangents to *γ*_*I*_  in *S*_*i*_  and *A*_*i*_ respectively.
- Global flexion angle *β*_*i*_ = *t*_*Si*_ + *t*_*Ai*_.

All parameters characterizing the shape of each flexed region are calculated. But main parameters representing the geometric structure of spine, pelvis in upstanding patients, are presented.

The pelvis in erected patient is represented by two parameters.

- One parameter represents its morphology: the pelvic incidence.
- One parameter describes its orientation in erected posture: the pelvic tilt.

The spinal structure is first characterized by the number of its plane flexed regions. Each regional plane is located using the two angles (*ψ* axial rotation about *Z*_0_ and *θ* lateral flexion about *X*_0_) locating its normal vector. The limits of each region are identified by their vertebral level. The global flexion *β* describes the regional curvature of the plane curve *γ*. All presented results show frontal, sagittal and horizontal projections of the 3D spinal curve with its flexed regions and zones of transitions. Tables of main parameter values are associated to the design of the spinal structure.

## 4. Results

**Figure 1:**
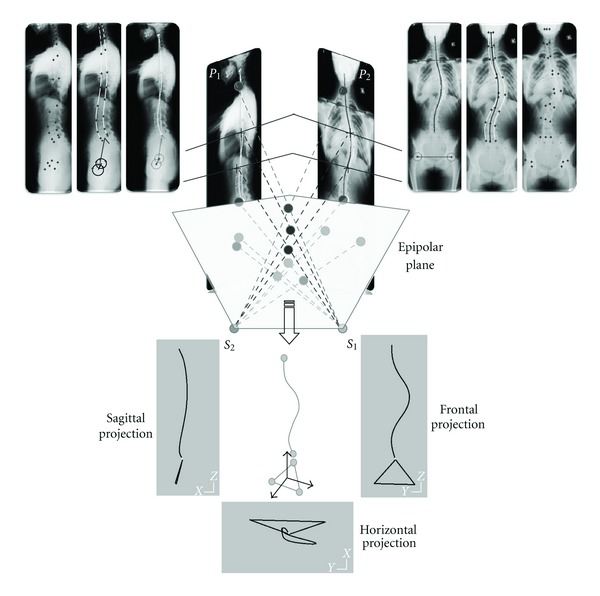
The techniques involved in the calculation of the 3D spinal curve from biplanar radiography coupled with photogrammetric reconstruction.

The goal of this paper is to describe the geometric structure of 3D spinal curves assumed to be previously calculated.  Figure 1 illustrates the technique used for accessing spinal curve from biplanar radiography, coupled with photogrammetric reconstruction.

The results of two different treatments are then compared. The first one assumes that the global spinal curve entirely belongs to a unique plane: the plane of maximum curvature. The second one detects several strictly plane regional sections along the spinal curve. Local geometric discontinuities are then exhibited. In asymptomatic subjects, the spinal structure is compound of three plane regions corresponding to lumbar, thoracic, and cervical curvatures. Short zones of transition are interposed between adjacent regions oriented differently. The modeling technique has been applied to patients suffering from low back pain. Local structural discontinuities of the spinal curve are observed. Scoliosis modifies significantly the spinal structure. An example of evolutive scoliosis is presented. Effects of implant on the spinal structure are shown.

### 4.1. Illustration of the New Technique

25 asymptomatic adults accepted a biplanar global examination (sagittal and frontal radiographic incidences). Photogrammetric reconstructions allowed to represent 3D models of pelvis and spinal curve. The pelvis is represented as a triangle based on femoral head centers and sacral plate center. 3D spinal curve and pelvic triangle are projected on sagittal plane, frontal plane, and horizontal plane. The spinal curve is compound of three plane regions and three zones of transition.

**Figure 2:**
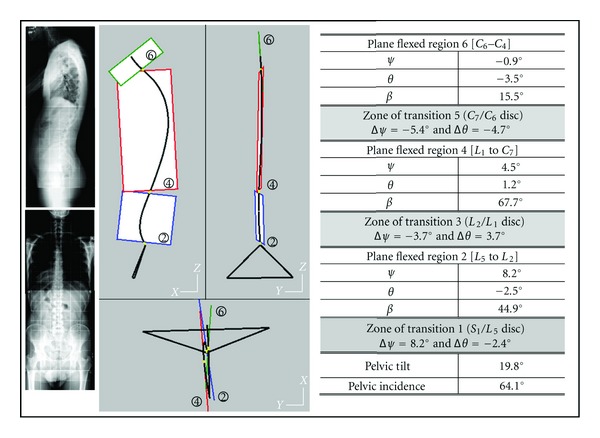
The technique allowing the access to the geometric structure of the spinal curve is illustrated by the results obtained with an asymptomatic subject (male 35 years). Are presented: the sagittal and frontal radiographic images, the sagittal, frontal, and horizontal global projections of the 3D spinal curve and the pelvic modeled as a triangle, the projections of the three plane regions, and the short zones of transition. A table gives numerical values: pelvic incidence PI and pelvic tilt PT, main parameters of the regional plane zones 2, 4, 6: components of the normal vector to the regional plane (*ψ* axial rotation about *Z*_0_, *θ* lateral flexion about *X*_0_), vertebral levels of regional limits, magnitude of the flexion angle bending the regional curve, main parameters of the zones of transition 1, 3, 5: vertebral levels of the zone, offsets of angular displacements (Δ*ψ* and Δ*θ*).

A case (male 35 years) is presented in Figure 2 the spinal curve is:

- zone 2 from *L*_5_ to *L*_2_: lumbar curvature,
- zone 4 from *L*_1_ to *C*_7_: thoracic curvature,
- zone 6 from *C*_6_ to *C*_4_: cervical curvature.

Three zones of transition are interposed between adjacent flexed regions:

- zone 1: *S*_1_/*L*_5_ disc,
- zone 3: *L*_2_/*L*_1_ disc,
- zone 5: *C*_7_/*C*_6_ disc.

The plane regions are characterized by the orientation of the normal vector to the regional plane (axial rotation *ψ* about *Z*_0_ and lateral flexion *θ* about *X*_0_) and by the angle *β* flexing the regional section of the spinal curve.

Angular offsets between normal vectors to adjacent regional planes: Δ*ψ* (axial rotation offset) and Δ*θ* (lateral flexion offset) move adjacent regional planes at level of zones of transition. When offsets are small, the zone of transition is restricted to a unique intervertral disc.

### 4.2. Geometric Spinal Structure Described Using Two Different Techniques

These techniques locate the unique plane of maximum curvature and the heterogeneous structure of spine compound of flexed regions and zones of transition.

**Figure 3:**
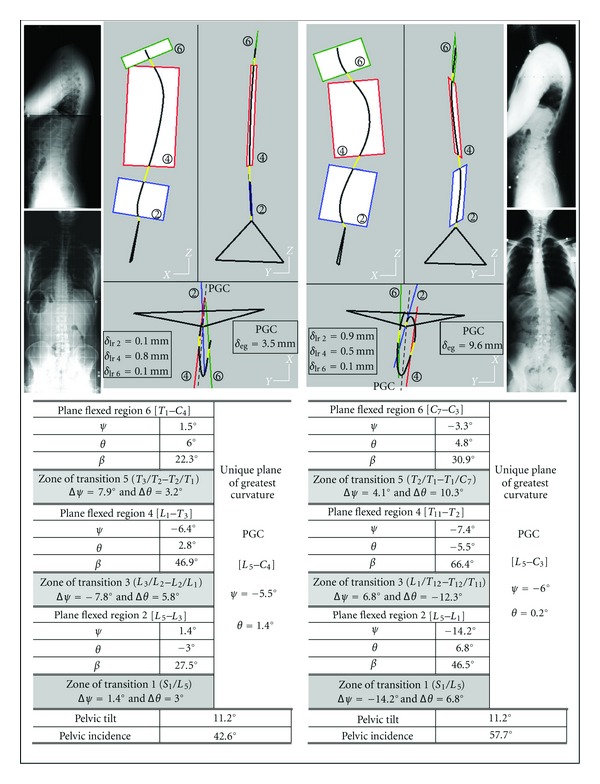
Cases of two asymptomatic adults (40 and 30 years). The comparison between modeling the spinal curve structure using a unique plane of greatest curvature PGC and the model involving regional planes and zones of transition. For each asymptomatic subject, are drawn: the PGC and the three regional planes and the zones of transition. The two PGCs have similar positions, but the regional structures are strongly different.

Two examples of asymptomatic subjects are presented (Figure 3) for illustrating the information brought by each of the two techniques:

- the plane of greatest curvature (PGC) is characterized by the orientation of the global normal vector to the plane (angles *ψ* about *Z*_0_ and *θ* about *X*_0_). The linear fitting coefficient of the rough global curve to the PGC is represented by the maximum normal distance *δ*_eg_ from 3D curve to PGC,
- the regional planes are represented by the orientations of their normal vector (*ψ* and *θ*) and by regional fitting linear *δ*_lr _and angular *δ*_ar_ coefficients. They represent linear and angular maximum offsets between 3D curve and regional planes.

The presented asymptomatic subjects have different spinal curve features: their PGC is not significantly rotated versus the sagittal plane *OX*_0_ *Z*_0_. In each case, the three regional planes are oriented differently, and their limits are different. The angular contribution of zones of transition may explain that these zones are lengthened.

### 4.3. Spinal Feature of a Patient Suffering from a Low Back Pain

**Figure 4:**
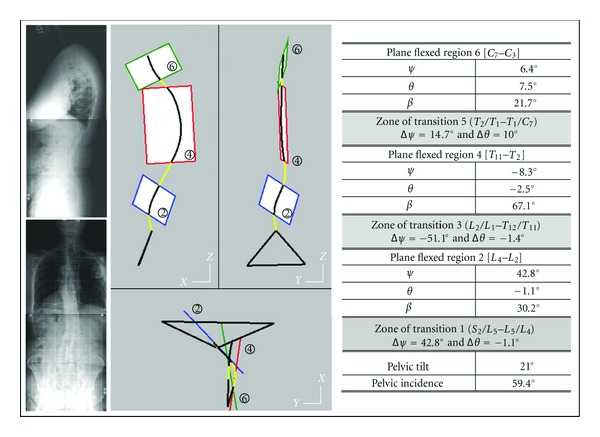
Patient suffering from a low back pain. The geometric structure is characterized by a strong rotation of the spinal lumbar plane plane region and by a correlative increasement of the sizes of zones of transition.

90 patients suffering from low back pain accepted a biplanar radiographic examination.  Figure 4 presents radiographic images of an adult male (60 years old) suffering from low back pain after a long practice of different sports (rugby, golf). The flexed region corresponding to the lumbar curvature is strongly rotated with respect to the anatomical sagittal plane. High level of axial rotation offsets between lumbar curvature versus sacrum and lumbar curvature versus thoracic one are distributed along zones of transition (*S*_1_/*L*_4_ and *L*_2_/*T*_11_, resp.) which include several intervertebral discs. The local heterogeneity of the spinal structure may be related to the patient pathology.

### 4.4. Scoliotic Patients

#### 4.4.1. The Spinal Structure Deformations due to the Pathology (Figure 5)

The radiographic file of an adult patient is presented for illustrating different effects of the pathology on the structure of the spinal curve. The whole spinal structure is strongly rotated versus the patient sagittal plane, main part of the rotation concerning the lumbar plane region. The thoracic curvature (from *L*_1_ to *C*_7_ in asymptomatic subject) is shared in two regions of opposite curvatures: *T*_11_–*T*_5_ and *T*_3_–*C*_3_. This last flexed region includes the cervical curvature. Zones of transition including several intervertebral discs are related to the high values of axial rotation of regional planes.

#### 4.4.2. The Structural Analysis of Evolutive Scoliosis (Figure 6)

A scoliotic patient accepted four biplanar radiographic examinations after orthopaedic treatment along a period of eight years (before, 2 years, 5 years, 8 years). The spinal structure keeps comparable global features versus time, but spinal deformations are related to pelvic tilting. After eight years, the set pelvis spine recovers its initial feature. It can be observed that regional planes slightly move between two successive radiographic examinations but corresponding flexion angles keep roughly constant. The regional curvatures are submitted to small rotations versus time, but flexed shapes are kept.

#### 4.4.3. Effect of a Surgical Operation Upon the Scoliotic Global Structure (Figure 7)

Three radiographic files describe the structural changes brought by a surgical operation: initial state, 6 months (before the surgical operation), and one year (after the operation).

The two first radiographic files show the structural evolution of the spinal curve before surgical operation: the pelvic tilt is the same, the lumbar regional plane slightly increases its axial rotation versus the patient sagittal plane. The angular offset between lumbar and thoracic regions is increased.

The surgical operation consisted in implanting a rod between *L*_4_ and *L*_1_, the zones of rod implantation corresponding to the limits of lumbar regional plane. The size of the lumbar regional curvature is reduced (from *L*_4_–*L*_1_, it becomes *L*_5_–*L*_3_). The zones of transition (where abduction and axial rotation are concentrated) are moved: before operation, they are *S*_1_/*L*_4_, *L*_1_/*T*_11_ and *T*_4_/*T*_2_, after operation, new values are *S*_1_/*L*_5_, *L*_3_/*L*_1_, *T*_3_/*T*_1_.

The surgical operation reduces significantly the axial rotation value of the lumbar region (28.4° versus 68.9°). Axial rotation offsets between adjacent plane regions strongly decrease: from −24.1° to −3.9° for the lumbar/thoracic transition zone and from −24.5° to −6.9° for the zone of transition between thoracic and cervical regions.

The surgical operation acts directly upon the orientation of the lumbar region, however the lumbar flexion angle is roughly constant. This operation has indirect effects upon the global spinal structure: initial discontinuities between plane regions are strongly decreased.

## 5. Discussion

In perfectly balanced subjects, spinal curves belong entirely to the anatomical sagittal plane. Lumbar, thoracic, and cervical curvatures are bounded by points of inflexion of the plane spinal curve. Our objective was to describe the spinal geometric structure of spines weakly or strongly deformed.

The mathematical representation of spinal curves is a prerequisite for accessing this goal. In the present study, it involved the access to radiographic systems giving images of the motionless patient shot under different incidences. The radiographic technique is coupled with photogrammetric reconstruction of points from their two images. Spinal curves are defined from series of isolated points.

Plane regions are detected along the rough spinal curve. This detection requires two successive steps, the first one locates its position, the second one eliminates aberrant points and restrict the regional extent so that two criteria would be satisfied. The first one checks that linear distances between points of the spinal curve and facing plane are lower than a maximum value. The second criterion verifies that planes passing through three adjacent points along the curve are very closed to the regional plane. The regional normal vector to the regional plane is calculated. The rough spinal curve facing each regional plane is projected on this plane. The regional plane projection of the 3D rough curve is uniquely flexed. The flexion angle bending this region is then obtained.

Zones of transition between two adjacent plane regions must transmit the angular offsets between the two plane orientations.

Directions of normal vectors to regional planes are related to anatomical axes (fixed axes) using two angles. The normal vector direction is obtained from two successive rotations moving the transverse axis *Y*_0_ to its actual location: one axial rotation about the fixed vertical axis *Z*_0_  followed by one rotation about the fixed posteroanterior axis *X*_0_. This solution has been preferred to the Euler sequence, because it involves rotations about moving axes. The rotation values about fixed axes may be licitly used when comparing the different orientations of regional planes for each examination so as their evolution versus time.

Geometric structures of spinal curves have been first estimated using different techniques. Experimental testing demonstrated the existence of an optimal radiographic incidence: the spinal curve shows its greatest curvatures when projected on the plane perpendicular to this specific incidence [17]. The plane of greatest curvature (PGC) has been then defined mathematically [14]. The presented technique best fits planes to local sections of the global spinal curve. The fitting calculation is based on the same principle as used for locating the plane of greatest curvature PGC: it minimizes the normal distance value between rough curve and fitted plane. A criterion of continuous planeity completes, in regional plane technique, the effects of the linear distance minimization. It was impossible to apply this criterion of continuous planeity to the global spinal curve. At level of points of inflexion, between opposite curvatures, local planes passing through three next points of the spinal curve are inaccurately located. This phenomenon is eluded with the technique of regional planes because changes in regional plane orientations are located at level of zones of transition.

The results show that asymptomatic subjects have closely oriented regional planes. The drawing of a unique plane of greatest curvature PGC may be licitly proposed. In other asymptomatic subjects, the proposal of a unique plane of greatest curvature is not obvious. The 3D location of unknown plane PGC is always possible, but linear distances between spinal curve and PGC may reach high values, not consistent with an accurate geometric modeling. The proposed geometric model of the spinal structure displays its heterogeneity. Even in asymptomatic subject, some regional sections of the spinal curve belong to planes oriented differently. In these regions the spinal curve is purely flexed. Axial rotation and abduction components due to the different orientations of adjacent regional planes are concentrated at level of zones of transition. These zones are restricted to a unique intervertebral disc when the adjacent regional planes are close together. They are longer in patients showing high regional discontinuities.

The proposed model has been applied to asymptomatic subjects. Local unbalances are displayed. It has also been tested with patients suffering from back pathologies, chronic low back pain and scoliosis. These pathologies entail spinal structure deformations. Geometric description of spinal curve features may allow clinical people to propose well-adapted correcting techniques.

A large diffusion of this technique involves the access to the mathematical representation of the spinal curve. Local solutions have been found from using biplanar radiography with successive or simultaneous exposures coupled with photogrammetric reconstructions. But this modeling technique may be adapted to any imaging system giving the accurate representation of spinal curves.

## Figures and Tables

**Figure 5:**
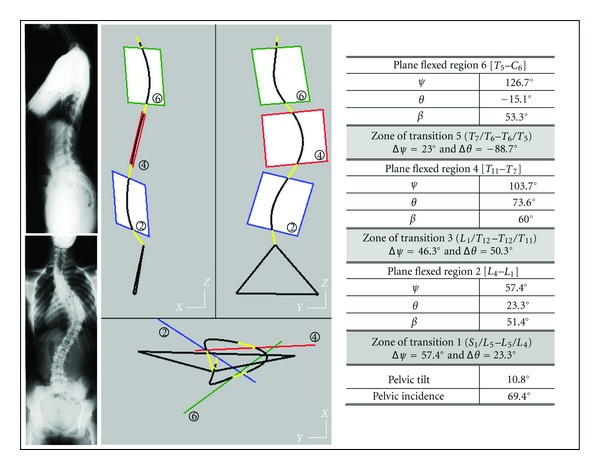
The scoliosis alters significantly the geometric structure of the spine.

**Figure 6:**
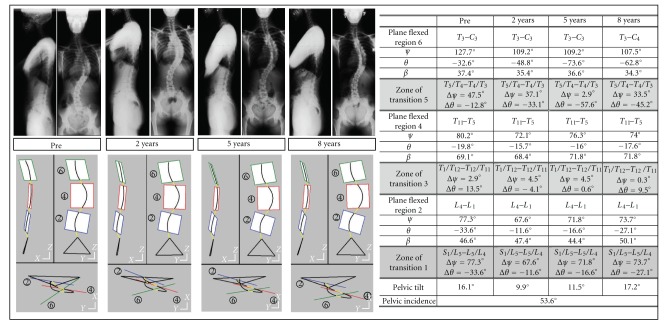
The different steps of scoliotic evolutions. The new technique allows medical doctors to quantify local effects of deforming pathologies.

**Figure 7:**
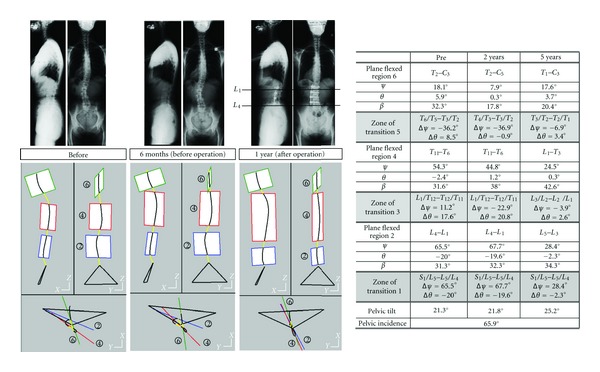
Illustration of the effects of a surgical operation upon a deformed spine.

## References

[1] Jackson RP, Peterson MD, McManus AC, Hales C (1998). Compensatory spinopelvic balance over the hip axis and better reliability in measuring lordosis to the pelvic radius on standing lateral radiographs of adult volunteers and patients. *Spine*.

[2] Bernhardt M, Bridwell KH (1989). Segmental analysis of the sagittal plane alignment of the normal thoracic and lumbar spines and thoracolumbar junction. *Spine*.

[3] Vedantam R, Lenke LG, Keeney JA, Bridwell KH (1998). Comparison of standing sagittal spinal alignment in asymptomatic adolescents and adults. *Spine*.

[4] Goldberg MS, Poitras B, Mayo NE, Labelle H, Bourassa R, Cloutier R (1988). Observer variation in assessing spinal curvature and skeletal development in adolescent idiopathic scoliosis. *Spine*.

[5] Boulay C, Tardieu C, Hecquet J (2006). Sagittal alignment of spine and pelvis regulated by pelvic incidence: standard values and prediction of lordosis. *European Spine Journal*.

[6] Berthonnaud E, Dimnet J (2007). Analysis of structural features of deformed spines in frontal and sagittal projections. *Computerized Medical Imaging and Graphics*.

[7] Berthonnaud E, Dimnet J, Roussouly P, Labelle H (2005). Analysis of the sagittal balance of the spine and pelvis using shape and orientation parameters. *Journal of Spinal Disorders and Techniques*.

[8] Berthonnaud E, Dimnet J (2006). Fast calculation ofparameters ofscolioses infrontal view forclinical applications. *ITBM-RBM*.

[9] Suh CH (1974). The fundamentals of computer aided X ray analysis of the spine. *Journal of Biomechanics*.

[10] Brown RH, Burstein AH, Nash CL, Schock CC (1976). Spinal analysis using a three dimensional radiographic technique. *Journal of Biomechanics*.

[11] Le Bras A, Laporte S, Mitton D, De Guise JA, Skalli W (2002). 3D detailed reconstruction of vertebrae with low dose digital stereoradiography. *Studies in Health Technology and Informatics*.

[12] Dubousset J, Charpak G, Dorion I (2005). A new 2D and 3D imaging approach to musculo-skeletal physiology and pathology with low-dose radiation and the standing position: the EOS system. *Bulletin de l’Academie Nationale de Medecine*.

[13] Andre B, Dansereau J, Labelle H (1994). Optimized vertical stereo base radiographic setup for the clinical three-dimensional reconstruction of the human spine. *Journal of Biomechanics*.

[14] Berthonnaud E, Dimnet J, Hilmi R (2009). Classification of pelvic and spinal postural patterns in upright position. Specific cases of scoliotic patients. *Computerized Medical Imaging and Graphics*.

[15] Berthonnaud E, Moyen B, Dimnet J (2000). Stereoradiographic techniques applied to three-dimensional clinical measurements. *Automedica*.

[16] Stagnara P, De Mauroy JC, Dran G (1982). Reciprocal angulation of vertebral bodies in a sagittal plane: approach to references for the evaluation of kyphosis and lordosis. *Spine*.

[17] Peloux J, Fauchet R, Faucon B, Stagnara P (1965). Le plan d'élection pour l'examen radiologique des cypho-scolioses. *Revue de Chirurgie Orthopedique et Reparatrice de l'Appareil Moteur*.

